# Panhypopituitarism and Central Diabetes Insipidus Almost Three Decades After Russell’s Viper Envenomation: A Remarkable Case Report and Literature Review

**DOI:** 10.18103/mra.v10i10.3195

**Published:** 2022-10-31

**Authors:** Ritwik Ghosh, Moisés León-Ruiz, Dipayan Roy, Sona Singh Sardar, Srijit Bandyopadhyay, Kunal Bole, Souvik Dubey, Julián Benito-León

**Affiliations:** 1 Department of General Medicine, Burdwan Medical College, and Hospital, Burdwan, West Bengal, India; 2 Section of Clinical Neurophysiology, Department of Neurology, University Hospital “La Paz,” Madrid, Spain; 3 All India Institute of Medical Sciences (AIIMS), Jodhpur, Rajasthan, India; 4 Indian Institute of Technology (IIT), Madras, Tamil Nadu, India; 5 School of Humanities, Indira Gandhi National Open University, New Delhi, India; 6 Department of Neuromedicine, Bangur Institute of Neurosciences, Institute of Post Graduate Medical Education and Research & SSKM Hospital, Kolkata, West Bengal, India; 7 Department of Neurology, University Hospital “12 de Octubre”, Madrid, Spain; 8 Centro de Investigación Biomédica en Red Sobre Enfermedades Neurodegenerativas (CIBERNED), Madrid, Spain; 9 Department of Medicine, Complutense University, Madrid, Spain

**Keywords:** Panhypopituitarism, central diabetes insipidus, Russell’s viper envenomation

## Abstract

**Background:**

Snakebite is a preventable yet often-neglected public health hazard with high chronic disability and mortality, mainly faced by rural communities in the tropics/subtropics. Endocrinological disorders following snakebite (especially Russell’s viper in India) are notably underrecognized and can lead to remarkable morbidity, poor quality of life, and cardiovascular mortality. Anterior pituitary insufficiency has been the most common ailment following Russell’s viper envenomation amid those endocrinological dysfunctions. On the contrary, the posterior pituitary and nearby hypothalamus mostly remain unharmed, so central diabetes insipidus is extremely rare following a viperid snakebite envenomation.

**Case Presentation:**

The authors present a patient developing panhypopituitarism with evident spontaneous central diabetes insipidus 29 years after Russell’s viper envenomation. Relevant investigations ruled out other possible etiologies, and he responded well to hormonal replacement therapy.

**Conclusions:**

Panhypopituitarism with concurrent central diabetes insipidus may occur following snakebite (especially in Russell’s viper envenomation). Early recognition and proper management of these complications are quintessential to preventing further misdiagnosis, under-recognition, morbidity, impaired quality of life, and mortality.

## BACKGROUND

Snakebite envenomation, a neglected, potentially salvageable, life-threatening medical emergency in tropical and sub-tropical countries, can cause severe multi-organ dysfunction (especially vascular, hematological, neurological, and muscular complications).^1,2^ Although they confer remarkable morbidity and mortality, endocrine dysfunctions following snakebite envenomation are under-reported and under-recognized because they often remain shrouded by other relatively severe and obvious issues, and the lack of clinical suspicion and diagnostic facilities in rural-based hospitals of tropical/sub-tropical developing countries. Amid these endocrine disorders, anterior pituitary insufficiency has been the most commonly detected manifestation following Russell’s viper envenomation (*Daboia russelii* in India; and *Daboia siamensis* in Burma).^3^ Russell’s viper envenomation-triggered hypopituitarism can manifest acutely (even during the initial admission for management of envenomation) or have a chronic or surprisingly delayed appearance, making the diagnosis further enigmatic.^3,4^ Neurohypophysis, in contrast to adenohypophysis, is resistant to vascular events (because it receives direct arterial supply from an inferior hypophyseal artery; and it tends to remain unaffected by intrasellar pressure changes during capillary leak syndrome and disseminated microthrombi formation). Therefore, Russell’s viper envenomation-mediated damage to neurohypophysis and resultant central diabetes insipidus is remarkably infrequent.^3,4^ Besides, concomitant hypocortisolism often can conceal manifestations of central diabetes insipidus, which get uncloaked only after corticosteroid treatment.^3^

The authors herein report an exceptional presentation of panhypopituitarism with central diabetes insipidus, 29 years after an event of Russell’s viper envenomation needing hospitalization, and review the relevant literature on this subject.

## CASE PRESENTATION

A 49-year-old previously healthy male from rural India (Burdwan, West Bengal) was brought to the emergency department with two episodes of generalized motor tonic-clonic seizures and confusion in the last 24 hours. After hemodynamic stabilization, his wife was called for detailed history taking. According to her, he complained of sluggishness of movements, generalized weakness, decreased appetite, increased thirst, increased daily urinary output, extreme loss of libido, and hoarseness of voice for the last six months. He had four hospitalizations for hyponatremic encephalopathy-like episodes in the previous four months. Past medical history was significant for a snakebite envenomation (identified as Russell’s viper by the emergency medical officer) 29 years back (in July 1993) needing hospitalization and infusion of anti-snake venom but no hemodialysis or ventilatory support. Otherwise, there was no other medical, traumatic, surgical, or recent medication history. He has been vaccinated against SARS-CoV-2 and has never contracted the disease in the last three years. He had normal growth and sexual development and was a father of two healthy adults.

Cognitive functions could not be tested as he was confused and drowsy with intermittent incoherent talks and bizarre behavior. He was afebrile with a normal respiratory rate and oxygen saturation but had tachycardia (114 bpm) and low systolic blood pressure (86 mmHg). Capillary blood glucose level was low (36 mg/dl). He was rapidly resuscitated with the infusion of intravenous thiamine and D50 solutions, but his consciousness level did not improve. Pertinent laboratory investigations were ordered, keeping the working diagnosis of metabolic encephalopathy due to glycopenia, hyponatremia, or prolonged post-ictal confused state (or non-convulsive status epilepticus). Complete blood cell count and renal and hepatic function tests were normal. Serum electrolytes revealed normal potassium, calcium, and magnesium levels but low sodium i.e., hyponatremia (116 mmol/L). During initial therapy, blood pressure, hypoglycemia, and sodium levels responded poorly to intravenous dextrose and normal saline infusions, for which vasopressor (norepinephrine) and 3% NaCl were added for maintenance of systolic blood pressure and sodium concentration, respectively. Suspecting an underlying hypopituitarism, relevant pituitary hormonal assays were ordered revealing an extremely low 8 A.M. serum cortisol level (1.0 μg/dL; biological reference range, 4.82–19.5 μg/dL), inappropriately low 8 A.M. plasma adrenocorticotropic hormone level (12.4 pg/mL; biological reference: 0.1–46.0 pg/mL), low free T3 (1.51 pg/mL; biological reference range 2.50–4.30), low free T4 (0.21 ng/dL; biological reference range 0.93–1.70), and inappropriately normal thyroid-stimulating hormone (2.542 μIU/mL; biological reference: 0.27–4.20 μIU/mL). Serum total testosterone level was low (0.8 ng/dL; biological reference range: 280–800 ng/dL), along with low insulin-like growth factor-1 (IGF-1) less than 15 ng/mL (reference: 57–241 ng/mL). Luteinizing hormone was 0.86 mIU/mL (biological reference interval: 1.7–8.6 mIU/mL), follicle-stimulating hormone was 2.14 mIU/mL (biological reference interval: 1.50–12.40 mIU/mL) and prolactin was 11.2 ng/mL (reference: 4.6–21.4 ng/mL).

**Figure 1:**
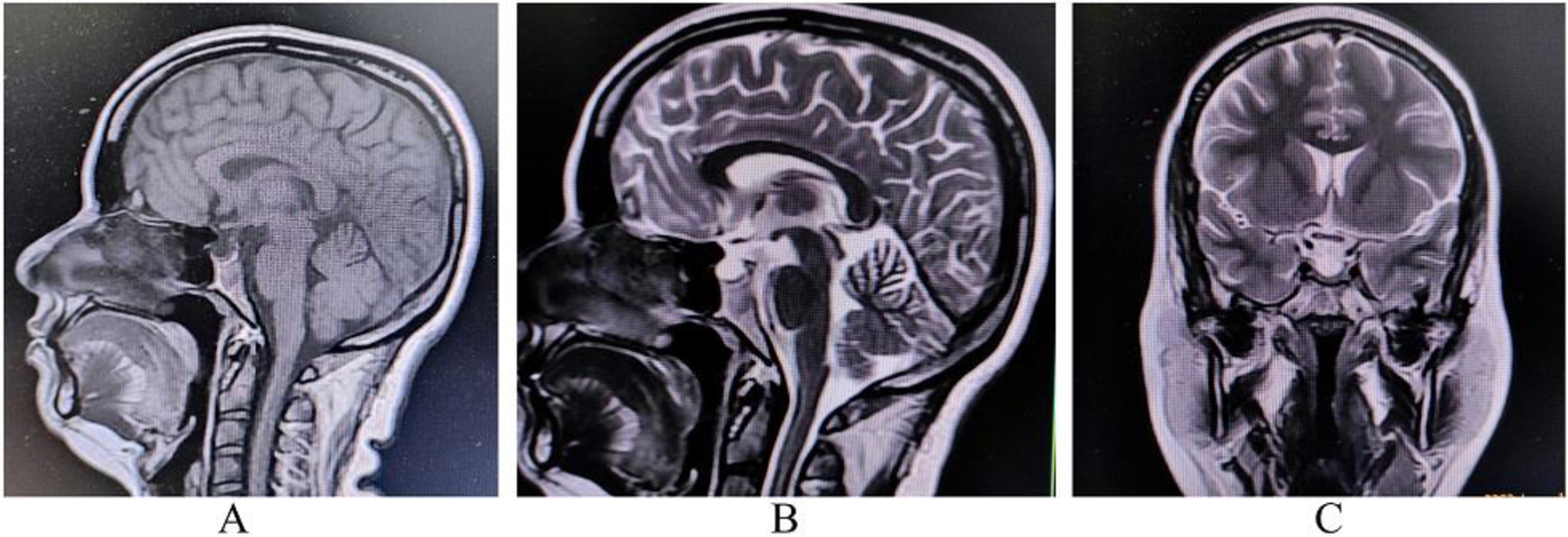
MRI of the brain and pituitary gland. Sagittal T1-weighted imaging (A), sagittal T2-weighted imaging (B), and coronal T2-weighted imaging (C) revealed a suprasellar cistern filled with cerebrospinal fluid and an absence of bright spot (a marker of posterior pituitary) on T1-weighted imaging (A) suggestive of complete empty sella sign.

The clinical and laboratory findings showed that the most tentative diagnosis was secondary adrenal insufficiency. Contrast-enhanced magnetic resonance imaging of the brain and pituitary demonstrated a thin and flat pituitary gland located on the floor of sella turcica with prominent cerebral spinal fluid space (Figure 1), compatible with empty sella syndrome. Intravenous hydrocortisone 100 mg three times a day was started from the third day of admission, followed by the addition of oral levothyroxine (50 mcg/day) from the eighth day of admission. Hyponatremia got smoothly corrected, and recurrent episodes of hypoglycemia abated as blood glucose levels stabilized after treating hypocortisolism. On day nine of admission, he was shifted to oral hydrocortisone (30 mg/day). However, symptoms of excessive thirst and polyuria had increased.

Urinalysis revealed no proteinuria or glycosuria, and the pH was 6.0. A 24-hour urine collection (without any fluid restriction) confirmed polyuria (3.6 liters/24 hours). The urinary excretions of uric acid, phosphate, calcium, citrate, and oxalate were also within normal limits. The serum osmolality was 302 mOsm/kg, but urine osmolality was 182 mOsm/kg. Serum copeptin levels could not be checked due to their unavailability in India. Hence, an inpatient modified water deprivation test confirmed the presence of central diabetes insipidus.

The bone density scan revealed severe vertebral osteoporosis (normal vitamin D level). An intramuscular testosterone enanthate injection (200 mg every three weeks) was initiated for hypogonadotropic hypogonadism. Meanwhile, for central diabetes insipidus, intranasal desmopressin, 10 μg twice daily (once at bedtime), was added alongside hydrocortisone, levothyroxine, calcium carbonate, and calcitriol. After six months of follow-up, there was a significant improvement in symptoms and general well-being. The impact of hormone replacement on his sexual life needs to be seen in further follow-ups.

## DISCUSSION

Although the exact pathological mechanisms of Russell’s viper envenomation-mediated pituitary insufficiency remain elusive, it has been postulated to be involving a three-step process. Step 1: enlargement/engorgement of the pituitary gland with inadequate vascular supply, making it susceptible to vascular insults, (due to capillary leak syndrome that occurs in Russell’s viper envenomation)^4^ direct venom-induced dose-dependent stimulation of endocrine cells and release of pituitary hormones.^5^ Step 2: a vascular insult to the vulnerable gland (supply-demand mismatch of the pituitary vasculature due to intrasellar and intravascular pressure changes),^4^ microthrombi deposition, or severe bleeding due to Russell’s viper envenomation-generated disseminated intravascular coagulation, circulatory shock, and intracranial hypertension.^6^ Step 3: resultant pituitary apoplexy (hemorrhage or necrosis) and insufficiency. In addition, autoantibody-mediated slow destruction of the pituitary gland resulting in the development of delayed pituitary insufficiency may be another explanation.^4^

Central diabetes insipidus eventuates only when almost 80–90% of arginine-vasopressin-producing magnocellular neurons of the hypothalamus get defunct. The role of neurohypophysis is only to store and secrete arginine-vasopressin and not to synthesize it; hence, for the genesis of central diabetes insipidus, the hypothalamus must be significantly involved.^7^ As far as we know, this would be the third reported case (Table 1) developing panhypopituitarism with concurrent spontaneous central diabetes insipidus (not induced by treatment with corticosteroids or associated underlying acute myocardial infarction or following SARS-CoV-2 infection) after Russell’s viper bite.^8–11^ There are few other cases of Russell’s viper envenomation-mediated central diabetes insipidus with panhypopituitarism; however, in those cases, central diabetes insipidus developed only after treating hypocortisolism with corticosteroids (without coexisting hypopituitarism).^12–14^

Russell’s viper envenomation-mediated pituitary insufficiency can eventuate abruptly within a few days of sustaining snakebite or be delayed for 24 years.^3^ Diagnostic delay can be attributed to a lack of clinical suspicion among clinicians, low snakebite-related health awareness amid the general population, scarcity of standard endocrinological setups even in tertiary-care centers, arbitrary course and non-specific symptomatology (i.e., fatigue, weight loss, anorexia, loss of libido, mood changes, and amenorrhea) of the disorder. Erstwhile, it is worth mentioning that the mean time to diagnose pituitary insufficiency post-Russell’s viper envenomation was 8.1 ± 3.6 years, according to an Indian study.^15^ However, our case is possibly the longest delay (i.e., 29 years). The delayed manifestation of panhypopituitarism with central diabetes insipidus resulted from occult pituitary damage during initial envenomation in 1993; unfortunately, it was not considered until date. Patients with Russell’s viper envenomation require long-term follow-up to identify delayed endocrine dysfunctions.^15^

Most studies agree that acute kidney injury following envenomation can strongly predict hypopituitarism.^12,14^ Most previously reported cases with panhypopituitarism and central diabetes insipidus had severe acute kidney injury needing hemodialysis.^8,9,12–14^ However, in our case, though the patient required 30 vials of anti-snake venom therapy, he did not develop acute kidney injury. The Myanmar Snakebite Project was a prospective study conducted for one year by the Australian government in collaboration with the Myanmar (Burma) government to improve outcomes for snakebite patients. Nine hundred forty-eight patients were included for analysis. Russell’s viper bites were responsible for all fatalities (9.8% of cases) and all cases of acute kidney injury. Panhypopituitarism was the most infrequent complication (2.1%).^16^

## CONCLUSIONS

In closing, snakebite envenomation can occasionally result in rare but serious atypical complications.^1,17,18^ Panhypopituitarism with concurrent central diabetes insipidus may occur following snakebite (especially in Russell’s viper envenomation). Early recognition and proper management of these complications are quintessential to preventing further morbidity, impaired quality of life, and mortality.

## Figures and Tables

**Table 1:** Summary of clinical and outcome data of reported cases with chronic panhypopituitarism, including central diabetes insipidus following Russell’s viper bite. Adapted from Antonypillai CN et al. 2011.^4^

Author and region	Russell’s viper subtype	Sex	Age at diagnosis (yrs)	Acute complications	Time to diagnose	Panhypopituitarism clinical features	Deficient hormone axes	Treatment	Outcome
Kolkata, West Bengal (India)^8^	Not reported	Male	20	Acute kidney injury, coagulopathy, and arterial hypotension	Eight years	Growth retardation and polyuria	Steroid, thyroid, gonadal, prolactin, insulin-like growth factor-1 and antidiuretic hormone	Prednisolone, levothyroxine, testosterone desmopressin, calcium carbonate and calcitriol	Well
Thrissur, Kerala (India)^9^	Not reported	Male	49	Acute kidney injury and dehydration	Four months	Diarrhea, vomiting, altered level of consciousness, fatigue, and polyuria	Steroid, thyroid, and antidiuretic hormone	Desmopressin	Well
The present case, 2022. Burdwan, West Bengal (India)	Daboia russelii	Male	49	Local swelling and regional lymphadenopathy	29 years	Generalized motor tonic-clonic seizures, confusion, sluggishness of movements, generalized weakness, anorexia, increased thirst, polyuria, extreme loss of libido, and hoarseness of voice	Steroid, thyroid, gonadal, and antidiuret ic hormone	Hydrocortisone, levothyroxine, testosterone desmopressin, calcium carbonate and calcitriol	Well

## Data Availability

The data supporting the findings of this study are available within the article.

## Availability of data and materials

Data sharing does not apply to this article as no data sets were generated or analyzed during the current study.
